# Serum Hypoalbuminemia Is a Long-Term Prognostic Marker in Medical Hospitalized Patients, Irrespective of the Underlying Disease

**DOI:** 10.3390/jcm11051207

**Published:** 2022-02-23

**Authors:** Howard S. Oster, Yardenna Dolev, Orli Kehat, Ahuva Weis-Meilik, Moshe Mittelman

**Affiliations:** 1 Department of Medicine A, Tel Aviv Sourasky Medical Center, Tel Aviv 6423906, Israel; yardennar@gmail.com; 2 Sackler Faculty of Medicine, Tel Aviv University, Tel Aviv 6997801, Israel; orlyk@tlvmc.gov.il (O.K.); ahuvawm@tlvmc.gov.il (A.W.-M.); 3 I-Medata AI Center, Tel Aviv Sourasky Medical Center, Tel Aviv 6423906, Israel

**Keywords:** albumin, hypoalbuminemia, prognosis, predictive model, mortality, hospitalization

## Abstract

Hypoalbuminemia is common in hypoalbuminemia-associated disorders (HAD), e.g., liver and kidney disease. We hypothesize that hospitalized patients with hypoalbuminemia have poor prognosis irrespective of their underlying disease. Records of patients admitted to Medicine (2010–2018), with and without HAD were analyzed, comparing low (<35 g/L) to normal serum albumin. Mann–Whitney and Chi-squared tests were used, and a logistic regression model was applied. Patients: 14,640 were admitted; 9759 were analyzed (2278 hypoalbuminemia: 736 HAD, 1542 non-HAD). All patients, and the subgroups with (as expected) and without HAD had worse outcomes. Specifically, in patients without HAD, those with hypoalbuminemia (*n* = 1542) vs. normal albumin (*n* = 6216) were older, had a higher Charlson Comorbidity Index (CCI, 5 vs. 4), longer median hospital stay (5 vs. 4), higher one year re-admission rate (49.9% vs. 39.8%), and one year mortality (48.9% vs. 15.3%, *p* < 0.001 for all). LR model predicting 3 month, 1 year and 5 year mortality confirmed the predictive power of albumin (1 year: OR = 4.49 for hypoalbuminema, *p* < 0.01). Hypoalbuminemia portends poor long-term prognosis in hospitalized patients regardless of the underlying disease and could be added to prognostic predictive models.

## 1. Introduction

Albumin is a water-soluble 65kD protein [1], synthesized by the liver [2]. It is the most abundant blood protein in humans, constituting about half of serum proteins, thus responsible for the oncotic pressure of the blood. The serum half-life of the molecule is approximately 20 days. Some of albumin’s main functions are binding non-soluble molecules in the serum and transporting medications and hormones [3].

The level of albumin considered as normal varies depending on the study. Most consider hypoalbuminemia as serum albumin levels lower than 34 or 35 g/L [4,5,6]. Several processes control plasma albumin concentration, including the absolute rate of albumin synthesis, the catabolic rate of the body, albumin distribution between the vascular and extravascular compartments and exogenous loss of albumin. The rate of albumin synthesis is affected by both nutrition and inflammation [7].

Hypoalbuminemia can be found in malnutrition (poor intake), advanced liver disease (impaired synthesis), kidney disease (increased loss), and in extreme catabolic states (increased breakdown) such as septicemia and metastatic carcinoma [3].

We have observed that hospitalized patients with hypoalbuminemia, regardless of their underlying disease tend to be sicker with a higher morbidity and mortality rate, than those with normal serum albumin levels. Occasionally, we see in the medical department two hospitalized patients with similar diseases, the same Charlson Comorbidity Index (CCI) [8], but the patient with hypoalbuminemia has a worse outcome.

The application of serum albumin level as a diagnostic or prognostic factor, outside the setting of hypoalbuminemia-associated disorders (HAD) has gained only little attention [9,10]. Here, we studied the role of serum albumin levels as an independent prognostic factor in hospitalized patients in the medical service. A distinction was made between patients with hypoalbuminemia-associated disorders (HAD) and other patients (non-HAD).

## 2. Patients and Methods

We reviewed the electronic charts of all (consecutive) patients admitted to the Department of Medicine A, Tel Aviv Sourasky Medical Center, a tertiary university hospital, between January 2010 and October 2018. Follow-up information was gathered until October 2019; 5-year mortality was assessed only for those admitted before October 2014. This study was approved by the Helsinki Committee (Institutional Review Board, IRB) of our institution.

Patients were included in the study if they had been admitted for at least 24 h to the inpatient wards of the department of internal medicine, were at least 18 years old and had at least one measure of serum albumin within the first 24 h.

Patients were excluded if they did not have any albumin measured at all, or if their albumin was measured after the first 24 h from the time of admission. If there had been more than one medical admission, only the data pertaining to the first admission were evaluated.

Hypoalbuminemia was defined as serum albumin levels lower than 35 g/L [4,5,6]. Patients with serum albumin level ≥35 g/L served as normal controls.

We used our hospital database and, using Structured Query Language (SQL) software, we searched the diagnostic codes (ICD-9) to identify the patients for this study. This was important for identifying the HAD vs. non-HAD diseases and also for computing the Charlson Comorbidity Index (CCI) [8]. For liver and kidney disease, we used a combination of the diagnostic codes and the elevation of liver function enzymes and creatinine respectively. For cancer, we searched for all diagnoses associated with cancer, carcinoma, neoplasm, tumor and malignancy and then manually chose the appropriate diagnoses that were malignant neoplasms. We then identified the subgroup where metastasis or metastatic was included in the diagnoses. For sepsis, we searched for any diagnosis with the word or part of the word sepsis or septic. We performed three separate analyses. In the whole group analysis, we evaluated all the patients meeting the inclusion criteria, comparing patients with hypoalbuminemia to those with normal serum albumin. Various parameters at hospital admission were compared, including age, Hb, serum creatinine levels and the CCI [8]. The assessed outcome values were length of stay (LOS) in the hospital, the percentage of patients staying in the hospital for more than 7 days (prolonged LOS), readmission rate within one year (yr), and finally the mortality rate within 1 yr.

We then applied a logistic regression model to predict mortality within 1 yr (also at 3 m and 5 yr), and assessed the effect of various parameters, including serum albumin, on mortality. 

In the HAD analysis, we focused on patients with hypoalbuminemia-associated disorders and made a similar comparison between patients with hypoalbuminemia and those with normal serum albumin. 

In the non-HAD analysis, we analyzed the mirror-image subgroup—patients with no HAD. After excluding those with HAD, a similar analysis, comparing patients with hypoalbuminemia to those with normal serum albumin was performed.

## 3. Statistical Analysis

For continuous variables we used Mann–Whitney U tests for comparison between groups. For categorical variables, Chi-squared test of independence was used for comparison between groups. 

To evaluate the relationship between levels of serum albumin in the first 24 h of hospitalization and mortality, a logistic regression model was applied. Albumin level was a categorical variable (cutoff of 35 g/L as defined above), other various clinical and laboratory parameters were the independent variables, while 1 yr (along with 3 m and 5 yr) mortality was the dependent variable. The predictive values of the models with and without the inclusion of serum albumin were assessed using the receiver operating characteristic (ROC) curve, comparing the area under the curve (AUC).

## 4. Results

**Figure 1:**
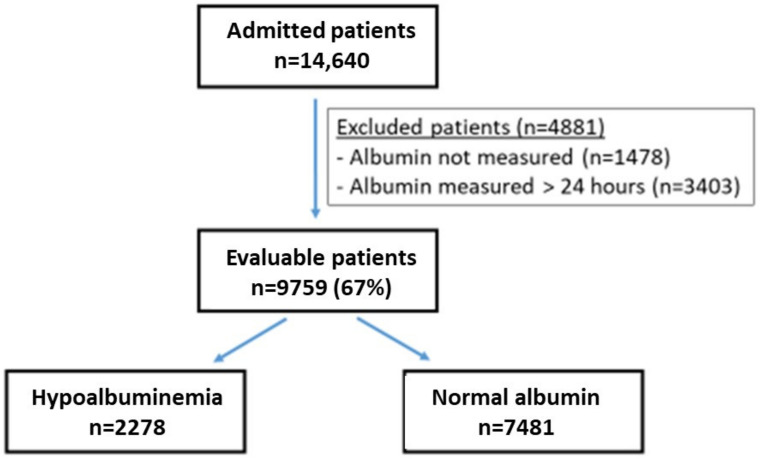
Patients in the study. Flow chart of patients included in the analysis. Two thirds of the admitted patients were evaluable. Among those, nearly 1/4 of the patients had low albumin levels upon admission. The criteria for excluding the 4881 patients were: no recorded albumin measure and albumin measured more than 24 h after the time of admission.

From 1 January 2010 through 10 October 2019, there were 14,640 patients admitted to the Department of Medicine A, an internal medicine service with 40 beds, at the Tel Aviv Sourasky Medical Center (Figure 1). Almost all of these patients had serum albumin tested, and 9759 (67% of the admitted) patients met the inclusion criteria and were evaluable. The patients who failed to meet the inclusion criteria (*n* = 4881) were excluded from the analysis, but in most relevant parameters resembled those who were included (data not shown). 

**Figure 2:**
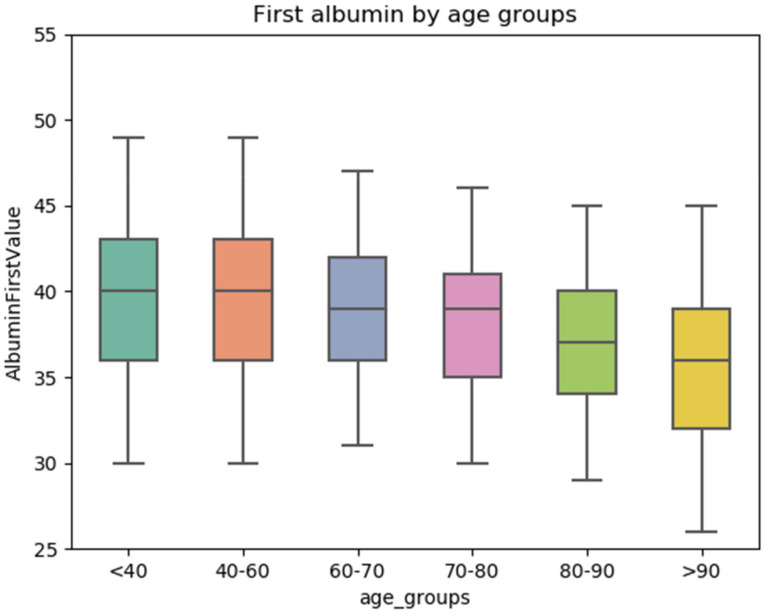
An inverse relation between serum albumin levels and age. Albumin stratified by age groups. After age 60, the average level of albumin begins to decline.

The whole studied group (*n* = 9759) included patients with hypoalbuminemia (*n* = 2278), and a control group of patients with normal serum albumin (*n* = 7481). Serum albumin was found to have an inverse relation with age (Figure 2). Table 1 summarizes patient characteristics. Patients with hypoalbuminemia were older (median 78.8 yr) than patients with normal serum albumin (72.4 yr, *p* < 0.001). There was no gender difference between the hypoalbuminemia and the control groups. Patients with hypoalbuminemia had significantly lower Hb level (median 109 vs. 128 g/L, *p* < 0.001), and higher serum creatinine level (136.1 vs. 93.7 µmol/L, *p* < 0.001) than the controls. The median Charlson Comorbidity Index (CCI), of patients with hypoalbuminemia was higher (median 5) than that of the controls (4, *p* < 0.001). Other routine lab values were not significantly different between the two patient subgroups, except for liver function abnormalities in some of the patients with hypoalbuminemia (data not shown). 

The whole group analysis resulted in several outcome differences between the two patient subpopulations (Table 2). The median hospital length of stay (LOS) was longer in patients with hypoalbuminemia than in the controls (5 vs. 3 days, *p* < 0.001), more patients with hypoalbuminemia had prolonged (>7 d) length of stay (LOS) in the hospital (31.3% vs. 15.2%, *p* < 0.001), and their hospital readmission rate within 1 yr was higher: 51.5% vs. 42.4% (*p* < 0.001). Finally, 1 yr mortality was significantly higher for patients with hypoalbuminemia, 51.0 vs. 17.4% (*p* < 0.001). 

**Figure 3:**
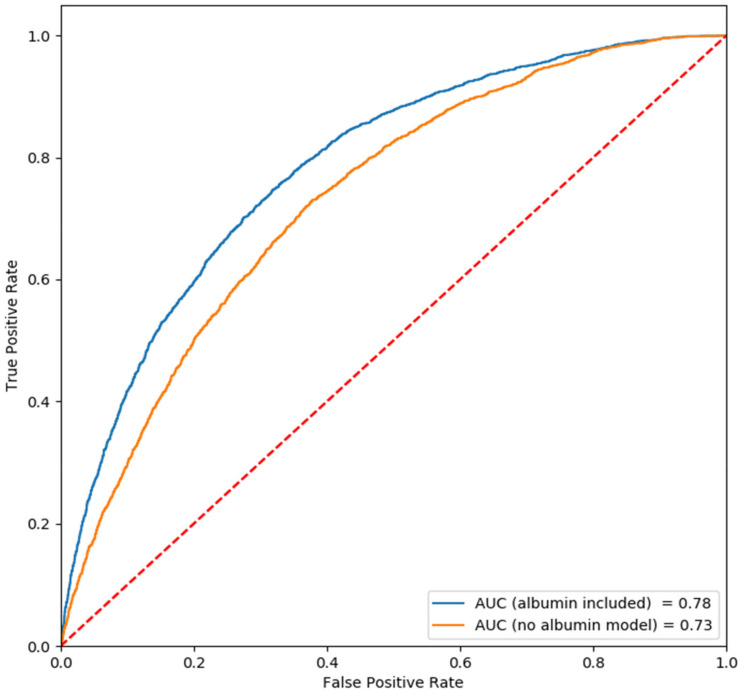
Whole group analysis—ROC curves (logistic regression, LR). ROC curve for the logistic regression model. Note that the AUC is improved when albumin is included in the model.

In the whole group analysis we also used a predictive logistic regression (LR) model and examined the contribution of serum albumin to this model. We initially included only age, gender and CCI (Table 3). Both age and the CCI were shown to be associated with 1 yr mortality, although the CCI, as expected, was more prominent (Figure 3; Table 3, lower region): CCI with OR = 1.27 (*p* < 0.01), age with OR= 1.02 (*p* < 0.01). The predictive value of this model, as expressed by the AUC curve, was 0.73. Adding serum albumin levels to the model improved its predictive value with AUC of 0.78. (OR = 4.19 for hypoalbuminemia, *p* < 0.01). As hypothesized, serum albumin level has inverse relation with prognosis. Additional parameters such as serum creatinine, bilirubin or Hb, failed to contribute to the predictive value of the model (data not shown). Replacing CCI with serum albumin in the LR model, using three parameters only, provided similar results (OR of hypoalbuminemia 4.5; AUC 0.77).

The results of an LR model to predict 3 m mortality, including age, gender, CCI and albumin, were similar to those of 1 yr mortality (Table S2, Supplementary Materials). All parameters, apart from gender were associated with 3 m mortality, with a predictive value, as expressed by the AUC, of 0.78. The main influencing factors were again CCI (OR = 1.24, *p* < 0.01), and hypoalbuminemia (OR = 4.14, *p* < 0.01).

An LR model to predict 5 yr mortality also provided similar results (Table S2, Supplementary Materials). Using age, gender, CCI and serum albumin in the model provided AUC of 0.79, with all parameters, except for gender, found to be associated with 5 yr mortality. Again, the more influential factors were CCI (OR = 1.23, *p* < 0.01) and hypoalbuminemia (OR = 5.03, *p* < 0.01).

It was important to find out whether serum albumin is an independent prognostic marker, or part of other disorders known to be associated with hypoalbuminemia (Hypoalbuminemia-associated disorders, HAD). As expected, patients with HAD (*n* = 2001) were older, had higher CCI, longer hospital LOS, higher re-admission rate in 1 yr and higher rate of 1 yr mortality than patients without disorders associated with hypoalbuminemia (*n* = 7758, Table S1, Supplementary Materials).

The HAD analysis focused on these patients (Table 4 and Table 5). This subgroup (*n* = 2001) included patients with liver diseases, kidney diseases, bacterial sepsis and metastatic cancer. The patients with HAD who actually had hypoalbuminemia (*n* = 736) were of similar age and had a similar CCI and 1 yr readmission rate as those with HAD and normal serum albumin (*n* = 1265). However, patients with HAD and hypoalbuminemia had longer hospital LOS (median 5 vs. 4 days), higher percentage of prolonged LOS (34% vs. 20.4%), and a significantly higher 1yr mortality (55.2% vs. 27.7%, *p* < 0.001), compared with HAD patients with normal serum albumin.

Applying the LR model to predict 1 yr mortality in HAD patients confirmed the power of age, CCI and albumin as predictors, with an OR for hypoalbuminemia of 3.40 (*p* < 0.01), and AUC for the model of 0.73 (Table 5). The AUC for both 3 m and 5 yr mortality was also 0.73. (Table S3 and Supplementary Materials).

The non-HAD analysis investigated the most relevant patient subpopulation, in whom HAD had been excluded (Table 6 and Table 7). Here, a similar investigation was performed. We compared the two patient subpopulations: non-HAD patients but with hypoalbuminemia (*n* = 1542), were older (78.2 vs. 70.7yr, *p* < 0.001), had higher CCI (5 vs. 4, *p* < 0.001), longer hospital LOS (5 vs. 3 days, *p* < 0.001), greater percentage of prolonged LOS (30.2% vs. 14.1%, *p* < 0.001), and a higher 1 yr re-admission rate (49.9% vs. 39.75%, *p* < 0.001) than non-HAD patients with normal serum albumin (*n* = 6216). Moreover, the 1 yr mortality rate was 48.9% in the non-HAD patients with hypoalbuminemia, as compared with 15.3% in the control group with normal serum albumin (*p* < 0.001, Table 6). The logistic regression model to predict 1 yr mortality in these non-HAD patients, including age, gender, CCI and albumin confirmed the strong effect of CCI (OR 1.26) and hypoalbuminemia (OR 4.49, *p* < 0.01), with AUC 0.79 (Table 7). 

The LR model to predict 3 m mortality in this patient population provided similar results, with CCI (OR= 1.26) and hypoalbuminemia (OR = 4.40, *p* < 0.01) as the influential factors and AUC of 0.79 (Table S4, Supplementary Materials). The results of the LR model to predict 5 yr mortality were consistent: CCI (OR = 1.28) and hypoalbuminemia (OR = 5.68) were predictors with AUC of 0.79 (Table S4, Supplementary Materials).

We also examined female and male patients separately. In the whole group the OR for hypoalbuminemia was 3.96 and 4.43 for women and men, respectively (*p* < 0.001 for both). 

**Figure 4:**
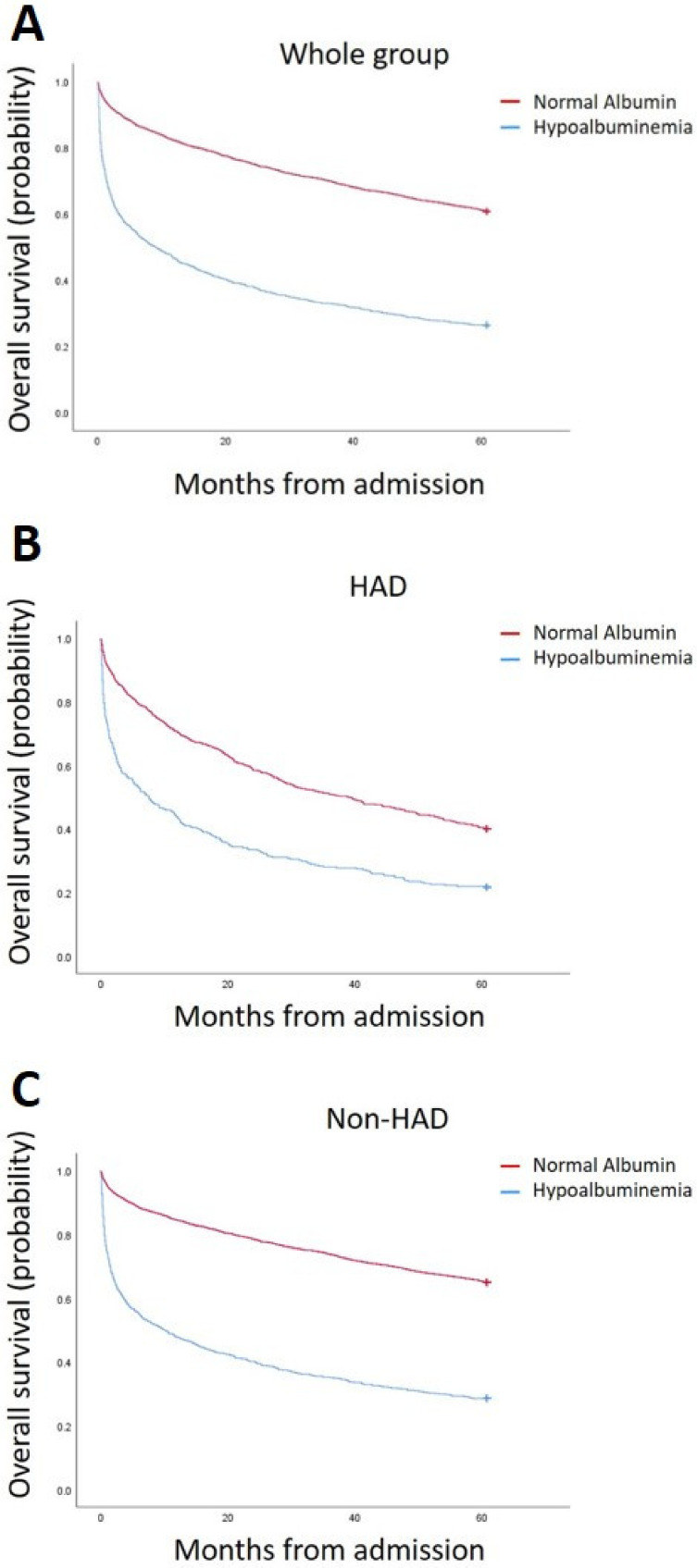
Kaplan–Meier curves comparing hypoalbuminemic patients to those with normal albumin. Panel (**a**) shows the curves for all patients; panel (**b**) for patients with hypoalbuminemia-associated disorders (HAD); and panel (**c**) for non-HAD patients. In all three cases, the average survival of the hypoalbuminemic patients was worse than that of those with normal albumin (*p* < 0.01).

The Kaplan–Meier survival curves for the whole group, the HAD patients and the non-HAD patients are demonstrated in Figure 4a–c, respectively. Note the dramatic difference in survival among patients with hypoalbuminemia vs. those with normal serum albumin in all three patient populations. This demonstrates that the albumin level can be a marker of disease severity irrespective of the underlying cause of illness.

We used cancer as another means to examine this independent association of low albumin and disease severity. Of all the patients, 15% had cancer, and of all those cancer patients, mortality was 50%. Subdivision of the cancer patients by albumin status demonstrated that the hypoalbuminemic patient mortality was 68% while the normal albumin patient mortality was 40%. The mortality of all non-cancer patients was lower: 20%. Hypoalbuminemia was associated with a mortality of 47%; normal albumin, only 14%.

## 5. Discussion

This study was based on clinical observations in the inpatient internal medicine wards and some previous studies, leading us to hypothesize that patients with hypoalbuminemia do worse than those with normal serum albumin, regardless of the underlying diseases or comorbidities. The main question asked for such patients is whether the initial level of albumin upon admission has prognostic significance. We tested this hypothesis and found that indeed albumin is an important independent prognostic predictor. 

Albumin has been shown to be a predictor in a number of studies in various settings. For example, some examine the albumin as a marker of nutritional status and its association with outcome [11] (e.g., death, length of hospital stay). Some look at mortality in specific settings: heart failure [12], renal cell carcinoma [4], ovarian cancer [13], and nasopharyngeal cancer [14]. There are also studies in the surgical setting after hip surgery [15]. Corti et al. examined over 4000 patients in an outpatient setting and found that reduced albumin was associated with increased mortality, especially in women [5], while Herrman et al. found that low serum albumin was a predictor of mortality in the 50% of internal medicine patients for whom albumin levels were measured [9]. In critical illness, the Acute Physiology and Chronic Health Evaluation (APACHE III) risk score includes serum albumin level and demonstrates a strong inverse correlation between serum albumin and mortality in such critically ill patients [10]. Owen et al. have shown that low serum albumin concentration in hemodialysis patients is a powerful predictor of death [16]. Not all agree on the association, however. Law et al. found no association between albumin level and mortality in over 21,000 men at a medical center in London [17], and a study in the Netherlands also found no significant association between albumin level and risk of cardiovascular disease or all causes of mortality [18]. Djousee found that low albumin was associated with increased mortality in women only [19]. Some studies examined the dynamics of the albumin level over time. Changes in albumin from pre- to post- surgery in patients with Crohn’s disease was useful [20], changes during the course of the first six months of peritoneal dialysis was significant as well [21], but in the above study from the Netherlands examining albumin changes over the course of three years, there was no effect on cardiovascular disease or mortality [18].

The uniqueness of the current study is its evaluation of albumin upon admission as a long-term prognostic indicator in hospitalized patients also after discharge from the medicine wards, and its examination of patients without hypoalbuminemia-associated disorders (HAD). 

Here, we show that hypoalbuminemia is associated with poor prognosis in these hospitalized patients. Hypoalbuminemia was found to be associated with older age, lower Hb level, higher serum creatinine level and higher CCI, as well as poor outcomes such as longer hospital stay, and higher re-admission rate. There was no difference in mortality based on gender. Moreover, patients with hypoalbuminemia were found in this study to have higher 3 m, 1 yr, and 5-yr mortality rates. A logistic regression (LR) model used to predict mortality in 3 m, 1 yr and 5 yr, confirmed the power of CCI as a predictor of mortality, and suggested that adding serum albumin to the model improves the predictive value. Interestingly, excluding the patients with HAD did not affect the power of the predictive model, suggesting that the role of serum albumin is independent and is unrelated to the underlying diseases.

Cancer is a disease with a high expected death rate (50% in our hospitalized patients), and mortality was higher in cancer patients with hypoalbuminemia (68% vs. 40%). The fact that the hypoalbuminemia patients had a higher mortality also in the non-cancer population (47% vs. 14%) is another demonstration that albumin is an independent predictor of disease severity. The role of hypoalbuminemia in HAD is relatively clear. For example, we would expect albumin to be low in situations where there is poor synthesis in liver disease, reduced intake in malnutrition, albumin loss in renal and gastrointestinal disease, and loss through increased vascular permeability in sepsis. Hypoalbuminemia is part of these diseases as well as a marker of their severity. We therefore also examined patients where such diseases were excluded, i.e., non-HAD. In patients without these diseases, the role of albumin is poorly understood. We know that albumin has important roles in human physiology: maintaining normal colloid osmotic pressure, binding and transport of minerals, hormones, proteins and medications, acting as an acid-base buffer and antioxidant, as well as enhancing immunologic status [18,22,23]. Its absence therefore should be detrimental to patients and as such, hypoalbuminemia may be not only a marker of disease severity, but also a part of the pathophysiology as well. If hypoalbuminemia is both a prognostic indicator as well as a part of the pathophysiology of disease, perhaps albumin administration might then be part of the treatment in such patients. Studies have been performed in a number of clinical settings. Patients with cirrhosis and spontaneous bacterial peritonitis benefit from the administration of albumin to prevent renal impairment and mortality [24]. Albumin administration also benefits cirrhotic patients with hepatorenal syndrome [25] and can reduce their systemic inflammation and immune suppression [26,27]. On the other hand, it was not beneficial in other settings like septic patients in the ICU [28,29,30].

The study has several limitations. First, it is retrospective which, to a certain extent, limits the comparison between groups. In addition, using diagnostic codes are limiting. For example, searching for metastatic disease yielded only 109 patients, lower than expected. Our chart reviews of patients with metastatic cancer revealed an appropriate code for a diagnosis of cancer in some patients, with appropriate details about metastasis in the text but no mention of metastasis in the official coded diagnosis. Despite this limitation, we used this method as an accepted and practical way to deal with a study of this size.

Although we would have liked to, we were unable to make an independent clinical assessment of the patients’ nutritional status. That a searching for “malnutrition” yielded only nine patients confirms the undercoding of this diagnosis. Moreover, because height is not usually measured in the inpatient ward setting, we had little reliable assessment of body mass index (BMI). 

Also, of the 14,640 admitted patients, approximately 1/3 of them were excluded either because albumin had not been measured at all or had not been measured within the first 24 h. We did, however, perform analyses on the 4881 excluded patients who had at least one albumin measurement and found that they too were similar to the 9759 patients included in our study (data not presented). Another limitation is that the analyzed patients were treated in a single institution, a tertiary care university hospital. Patients in community hospitals were not studied. However, the fact that our medical center is in the center of a large city provides heterogeneity (e.g., socio-economic, racial) which makes the study more robust and generalizable. We did not examine changes in albumin over time, which some studies found to be useful in improving the outcome predictions. The study was designed to answer the question as to whether, upon admission, one can make an assessment of prognosis given the information at hand at that time. Future work will examine the subset of patients who are able to increase their albumin over time and assess whether their prognosis improves as well. Finally, differentiation between the HAD and non-HAD patients was based on the recorded diagnoses in the medical records. However, we assume that any errors in charting would be similar in all groups, whether the patients have normal levels or low levels of albumin. 

## 6. Conclusions

In summary, this study demonstrates that in patients admitted to a department of internal medicine, serum albumin upon admission is an important long-term prognostic factor. Most important, this is true even in patients for whom hypoalbuminemia may not be an intrinsic part of their disease. Despite some mixed results in the literature and possible gender differences reported by others, our results are clear and pave the way to including albumin as part of prognostic scores such as the CCI. Future work will include assessing the long-term prognosis in hypoalbuminemic patients with both acute and chronic disease who manage to increase their albumin with time. 

## Figures and Tables

**Table 1:** Patient characteristics.

	All Patients	Hypoalbuminemia	Normal Albumin	*p*
*n*	9759	2278	7481	-
Age median (IQR *)	74.04 (60.0–84.1)	78.8 (65.4–87.6)	72.4 (58.6–83.0)	*p* < 0.001
Gender: Males	50.9%	50.53%	51.08%	-
Serum albumin g/L median; (IQR)	38 (3.6–4.1)	30.5 (2.9–3.3)	40 (3.7–4.2)	*p* < 0.001
Hb g/L, median; (IQR)	125 (109–138)	109 (95–124)	128 (115–134)	*p* < 0.001
Creatinine µmol/L; median; (IQR)	94.6 (76.9–123.8)	136.1 (74.3–151.2)	93.7 (79.6–117.6)	*p* < 0.001
CCI median (IQR)	4 (2–6)	5 (4–7)	4 (2–5)	*p* < 0.001

* IQR: Inter quartile range—percentile 25–75th; CCI: Charlson Comorbidity Index.

**Table 2:** Whole group analysis—comparison (unadjusted) between all patients with hypoalbuminemia and the control group (normal albumin).

	Hypoalbuminemia	Normal Albumin	*p*
*n*	2278	7481	
LOS (median) (IQR *)	5d (3–9)	3d (2–6)	*p* < 0.001
Prolonged stay (>7 days)	31.3%	15.2%	*p* < 0.001
Re-admission in 1 yr (%)	51.5%	42.4%	*p* < 0.001
1 yr Mortality (%)	50.97%	17.4%	*p* < 0.001

Abbreviations: LOS: Length of stay in the hospital (in days), * IQR: Inter quartile range (percentile 25–75th).

**Table 3:** Whole group analysis—logistic regression (LR) model predicting 1 yr mortality.

**3 Parameters**	**OR**	**95% CI**	***p*** **-Value**	**Prediction Value**
Age	1.02	1.02–1.03	<0.01	
Gender	0.99	0.90–1.10	0.89	
CCI	1.27	1.24–1.31	<0.01	
AUC				0.73
**4 Parameters**	**OR**	**95% CI**	***p*-Value**	**Prediction Value**
Age	1.02	1.02–1.03	<0.001	
Gender	1.00	0.90–1.11	0.97	
CCI	1.24	1.21–1.27	<0.01	
Hypoalbuminemia	4.19	3.76–4.67	<0.01	
AUC				0.78

**Table 4:** HAD analysis (unadjusted): Patients with hypoalbuminemia-associated disorders.

	Hypoalbuminemia	Normal Albumin	*p*
*n*	736	1265	
Age median (IQR *)	79.9 (68.2–87.4)	78.4 (67.2–85.8)	0.07
CCI median (IQR)	6 (5–8)	6 (5–8)	0.14
LOS median, days (IQR)	5 (3–9)	4 (2–7)	<0.001
Prolonged stay (%) (>7 days)	34%	20.4%	<0.001
Re-admission in 1 yr (%)	54.9%	55.3%	0.89
1-year Mortality (%)	55.2%	27.7%	<0.001

* IQR: Inter quartile range (percentile 25–75th); LOS: Length of stay in the hospital (in days).

**Table 5:** HAD analysis—logistic regression (LR) to predict 1 yr mortality in HAD patients.

	OR	95% CI	*p*	Prediction Value
Age	1.02	1.02–1.03	*p* < 0.01	
Gender	1.09	0.89–1.33	*p* = 0.43	
CCI	1.18	1.13–1.24	*p* < 0.01	
Hypoalbuminemia	3.40	2.78–4.16	*p* < 0.01	
AUC				0.73

**Table 6:** Non-HAD analysis (unadjusted)—patients without hypoalbuminemia-associated disorders.

	Hypoalbuminemia	Normal Albumin	*p*
*n*	1542	6216	
Age median (IQR *)	78.2 (64.8–86.9)	70.7 (56.4–82.1)	<0.001
CCI median (IQR)	5 (3–6)	4 (2–5)	<0.001
LOS median (IQR)	5 (3–8)	3 (2–5)	<0.001
Prolonged Stay (%) (>7 days)	30.2%	14.1%	<0.001
Re-admission in 1 yr (%)	49.9%	39.75%	<0.001
1 yr Mortality (%)	48.9%	15.3%	<0.001

* IQR: Inter quartile range (percentile 25–75th).

**Table 7:** Non-HAD analysis—logistic regression (LR) model predicting 1 yr mortality in patients in whom HAD was excluded.

	OR	95% CI	*p*	Prediction Value
Age	1.02	1.02–1.03	*p* < 0.01	
Gender	0.98	0.87–1.11	*p* = 0.73	
CCI	1.26	1.22–1.31	*p* < 0.01	
Hypoalbuminemia	4.49	3.95–5.12	*p* < 0.01	
AUC				0.79

## Supplementary Materials

The following supporting information can be downloaded at: https://www.mdpi.com/article/10.3390/jcm11051207/s1, Table S1: Patients with hypoalbuminemia-associated disorders (HAD) vs. those with no HAD (Non-HAD); Table S2: Logistic regression model for 3 month and 5 year mortality; Table S3. Logistic regression model for 3 month and 5 year mortality in HAD patients; Table S4. Logistic regression model for 3 month and 5 year mortality in non-HAD patients.
