# Supplementary material for: Serum Hypoalbuminemia Is a Long-Term Prognostic Marker in Medical Hospitalized Patients, Irrespective of the Underlying Disease
Source: J Clin Med. 2022 Feb 23;11(5):1207. doi: 10.3390/jcm11051207 (PMC8911288; doi:10.3390/jcm11051207)
Supplement: Supplementary file 1 [file jcm-11-01207-s001.zip › jcm-1551605-supplementary.pdf]

Supplement

Table S1. Patients with hypoalbuminemia-associated disorders (HAD) vs those with no HAD (Non-HAD).

	HAD	Non-HAD	P value.
**n**	**2001**	**7758**	
**Age** median (IQR*)	**78.9** (67.4-86.2)	**72.3** (57.9-83.3)	**P < 0.001**
**CCI** median (IQR)	**6** (5-8)	**4** (2-5)	**P < 0.001**
**Albumin** median g/L (IQR)	**36** (33-41)	**39** (35-42)	**P < 0.001**
**LOS** median (IQR)	**4** (3-8)	**3** (2-6)	**P < 0.001**
**Prolonged stay %** (> 7days)	**25.4%**	**17.3%**	**P < 0.001**
**Readmission in 1 yr** (%)	**55.1%**	**41.8%**	**P < 0.001**
**1-yr mortality** (%)	**37.8%**	**22.0%**	**P < 0.001**

Abbreviations: IQR: Inter quartile range (percentile 25th – 75th)**; CCI**: Charlson comorbidity index; **LOS**: Length of stay (in days).

Table S2. Logistic regression model for 3 month and 5 year mortality

Whole group analysis – logistic regression to predict 3m mortality

	OR	95% CI	P	Prediction Value
**Age**	**1.02**	1.02-1.03	< 0.01	
**Gender**	0.99	0.90-1.10	0.91	
**CCI**	**1.24**	1.21-1.27	< 0.01	
**Hypoalbuminemia**	**4.14**	3.73-4.60	< 0.01	
**AUC**				**0.78**

Whole group analysis – logistic regression to predict 5yr mortality

	OR	95% CI	P	Prediction Value
**Age**	**1.03**	1.02-1.03	**< 0.01**	
**Gender**	0.99	0.87-1.14	0.94	
**CCI**	**1.23**	1.18-1.28	**< 0.01**	
**Hypoalbuminemia**	**5.03**	4.32-5.86	**< 0.01**	
**AUC**				**0.79**

Table S3. Logistic regression model for 3 month and 5 year mortality in HAD patients

HAD analysis – logistic regression to predict 3m mortality

	OR	95% CI	P	Prediction Value
**Age**	**1.02**	1.02-1.03	p<0.001	
**Gender**	1.13	0.93-1.38	p=0.214	
**CCI**	**1.19**	1.13-1.24	p<0.001	
**Hypoalbuminemia**	**3.45**	2.84-4.20	p<0.001	
**AUC**				**0.73**

HAD analysis – logistic regression to predict 5yr mortality

	OR	95% CI	P	Prediction Value
**Age**	**0.04**	1.02-1.05	p<0.001	
**Gender**	0.93	0.70-1.23	p=0.60	
**CCI**	**1.12**	1.05-1.2	p<0.01	
**Hypoalbuminemia**	**3.51**	0.64-4.68	p<0.001	
**AUC**				**0.73**

Table S4. Logistic regression model for 3 month and 5 year mortality in non-HAD patients

Non-HAD analysis – logistic regression to predict 3m mortality

	OR	95% CI	P	Prediction Value
**Age**	**1.02**	1.02-1.03	p<0.001	
**Gender**	0.96	0.85-1.08	p=0.46	
**CCI**	**1.26**	1.21-1.30	p<0.001	
**Hypoalbuminemia**	**4.40**	3.88-4.99	p<0.001	
**AUC**				**0.79**

Non-HAD analysis – logistic regression to predict 5yr mortality

	OR	95% CI	P	Prediction Value
**Age**	**1.02**	1.02-1.03	p<0.001	
**Gender**	1.02	0.87-1.19	p=0.83	
**CCI**	**1.28**	1.21-1.35	p<0.001	
**Hypoalbuminemia**	**5.68**	4.74-6.80	p<0.001	
**AUC**				**0.79**
